# Structural abnormalities in erythrocytes and a potential denucleation process induced by metformin, compromising cell viability in specimens of *Astyanax lacustris* (Pisces, Characidae)

**DOI:** 10.1007/s11356-026-38072-5

**Published:** 2026-07-23

**Authors:** Luara Lupepsa, Pablo Américo Barbieri, Brennda Ribeiro Paupitz, Vanessa Oliveira Sales-Zampar, Helito Volpato, Fernanda Errero Porto Saparolli, Eliane Papa Ambrosio-Albuquerque, Carlos Alexandre Fernandes, Luciana Andreia Borin-Carvalho, Ana Luiza de Brito Portela-Castro

**Affiliations:** 1 Department of Biotechnology, Genetics and Cell Biology, State University of Maringá (UEM), Avenida Colombo, 5790, Maringá, Paraná CEP 87020‑900 Brazil; 2 Center for Human Sciences and Education, Collegiate of Biological Sciences Course, State University of Paraná (UNESPAR), Paranavaí, Paraná Brazil; 3 Department of Morphological Sciences, State University of Maringá (UEM), Maringá, Paraná Brazil

**Keywords:** Antidiabetic drug, Cytotoxicity, Ecotoxicology, Fish, Flow Cytometry, Genotoxicity

## Abstract

Hematological studies in fish are sensitive indicators of physiological and pathological changes caused by aquatic pollution, including pharmaceutical contaminants. Among these pollutants, metformin is one of the most prevalent and has been associated with adverse effects on aquatic organisms. However, its impact on fish erythrocyte morphology remains poorly understood. This study evaluated the cyto- and genotoxic effects of metformin on erythrocytes of *Astyanax lacustris* through morphological analyses, membrane integrity assessment by flow cytometry, and DNA damage evaluation using comet assay. Fish were exposed to metformin concentrations of 50, 100, and 10,000 µg/L for 90 days. Exposure induced significant erythrocyte alterations, particularly at higher metformin concentrations, including membrane invaginations and ruptures, cytoplasmic vacuolization, nuclear abnormalities, DNA damage, peripheral nuclei, and formation of erythroplastids, suggesting denucleation process. Flow cytometry also revealed an increased proportion of non-viable cells. These findings suggest that metformin may significantly impair erythrocyte physiology and viability, potentially compromising the health of exposed fish.

**Supplementary Information:**

The online version contains supplementary material available at 10.1007/s11356-026-38072-5.

## Introduction

Pharmaceutical products, which are extensively used worldwide, enter aquatic environments through several pathways, including human consumption, improper medication disposal, waste generated by the pharmaceutical and healthcare sectors, and excretion via urine or feces from both humans and animals (Blair et al. 2013; MacLaren et al. 2018; Hejna et al. 2022; Aziz et al. 2025). These contaminants pose ecological risks to non-target organisms and may also threaten human health when they contaminate drinking water sources (Hejna et al. 2022). Numerous reviews have reported the presence of pharmaceuticals in aquatic environments, particularly those with high consumption rates, such as antibiotics, analgesics, anti-inflammatory drugs, blood lipid regulators, steroid hormones, and antihyperglycemic agents, among others (Santos et al. 2021; Balakrishnan et al. 2022; Hejna et al. 2022; Zheng et al. 2024; Aziz et al. 2025).

Among antihyperglycemic agents, metformin stands out as one of the most frequently detected pharmaceutical contaminants in aquatic environments, with concentrations progressively increasing and posing potential ecological risks (Blair et al. 2013; MacLaren et al. 2018; Ussery et al. 2018, 2024; Balakrishnan et al. 2022; Zheng et al. 2024). This compound has been detected in aquatic environments across 91 countries, with reported concentrations ranging from 0.2 ng/L to 1180 μg/L (Zheng et al. 2024). Metformin demonstrates considerable stability in aqueous solutions at temperatures ranging from 30 to 70 °C, exhibiting only approximately 10% degradation over a period exceeding eight days, even under elevated temperatures (Sharma et al. 2010). In Brazil, Quadra et al. (2021) investigated four reservoirs located in the Amazon and Atlantic Forest regions and reported that metformin exhibited the highest concentrations among the pharmaceuticals analyzed, reaching 2.191 ng/L in one reservoir. This pharmaceutical has been detected in a wide variety of environments, including effluents from wastewater treatment plants (WWTPs), sewage treatment facilities, hospital sewage and sludge, surface waters (rivers, lakes, seas, and oceans), and even tap/drinking water (Zheng et al. 2024).

From a clinical perspective, metformin is recognized as a major antihyperglycemic agent and has been used worldwide for the treatment of type II diabetes mellitus for more than a century (Bailey 2017). Beyond its primary therapeutic use, experimental studies suggest that metformin may also provide beneficial effects in the management of other human disorders, including polycystic ovary syndrome, Parkinson’s disease, and Alzheimer’s disease (Dutta et al. 2023). Furthermore, metformin has been associated with a reduced incidence of cancer and, when combined with other therapeutic approaches, may contribute to lower cancer-related mortality rates (Adak et al. 2018; Saraei et al. 2019; Dutta et al. 2023).

Metformin is not fully metabolized by the human body and is excreted largely unchanged, contributing to its continuous release into aquatic environments (Graham et al. 2011). This persistence is further exacerbated by the limitations of conventional wastewater treatment technologies, which are often inefficient in removing metformin, thereby posing potential risks to aquatic ecosystems (MacLaren et al. 2018; Zheng et al. 2024). Although several methods have been proposed for the removal of metformin from contaminated water, their practical implementation remains limited due to factors such as complex production processes, generation of byproducts, and challenges associated with post-adsorption treatment (Balakrishnan et al. 2022; Pap et al. 2023).

Recent studies have considerably advanced our understanding of the environmental impacts of metformin, particularly its effects on non-target organisms such as fish. The effects of metformin on fish are complex, affecting reproductive performance, endocrine and genetic systems (including endocrine disruption), contributing to oxidative stress, and altering the expression of genes related to metabolism and detoxification (Niemuth and Klaper 2015; Niemuth et al. 2015; Crago et al. 2016; MacLaren et al. 2018; Lee et al. 2019; Sibiya et al. 2023). Metformin has also been shown to affect fish embryonic development, as demonstrated by Elizalde-Velázquez et al. (2021) in zebrafish (*Danio rerio*). In that study, zebrafish embryos exposed to different concentrations of metformin (1, 10, 20, 30, 40, 50, 75, and 100 μg/L) exhibited accelerated hatching and several morphological alterations associated with oxidative damage mechanisms. Considering the available literature, the experimental effects of metformin are substantial and ecotoxicologically relevant, regardless of the biomarker employed.

The erythrocytes of teleost fish exhibit morphological characteristics comparable to those of other non-mammalian vertebrates, such as amphibians, reptiles, and birds, typically presenting an elliptical to oval shape and, unlike mammalian erythrocytes, retaining a nucleus (Witeska 2013; Arnaudov and Arnaudova 2023). Vertebrates possessing nucleated erythrocytes serve as sensitive bioindicators of environmental pollution, frequently displaying nuclear abnormalities, including increased frequencies of micronuclei, which are considered important biomarkers of genotoxicity (Cherednichenko et al. 2024). In fish, erythrocyte morphology is adapted to support the rapid demands of tissues for oxygen and nutrients, making these cells valuable indicators for monitoring environmental stressors (Vázquez and Guerrero 2007). Although hematological studies in fish have been conducted for more than 70 years, they have recently gained increasing attention due to their effectiveness as sensitive indicators of physiological and pathological changes resulting from aquatic pollution (Arnaudov and Arnaudova 2023). Prolonged exposure to pharmaceutical contaminants may induce various alterations in fish erythrocytes, including membrane and nuclear deformities, micronucleus formation, amitosis, and hemolysis, thereby providing deeper insights into ecotoxicological effects (Witeska 2013; Cherednichenko et al. 2024).

Small characids belonging to the family Characidae, particularly species of the genus *Astyanax* Baird & Girard, 1854, exhibit one of the broadest geographic distributions among Neotropical freshwater fishes, inhabiting a wide variety of environments throughout the Neotropical region (Terán et al. 2020). Consequently, these species are particularly vulnerable to environmental contamination and are considered important bioindicators (Tincani et al. 2019). Among species of this genus, the yellowtail lambari *Astyanax lacustris* Lütken, 1875 has proven to be an excellent model for cytotoxicity and genotoxicity studies involving exposure to various contaminants, including heavy metals (Silva et al. 2020), pesticides (Rossi et al. 2020), and textile dyes (Silva et al. 2024), among others.

Due to the limited number of studies investigating the long-term effects of metformin using erythrocytes as biomarkers, the present study aimed to evaluate the cytogenotoxic potential of metformin in *A. lacustris* following chronic exposure to different concentrations of the drug. Blood cells (erythrocytes) were employed as biomarkers to assess exposure-related effects. We hypothesized that environmentally relevant concentrations of metformin could induce cytotoxic and genotoxic effects in the erythrocytes of *A. lacustris*, potentially compromising cellular viability.

## Material and methods

### Chemicals

Metformin (1,1-dimethylbiguanide hydrochloride) was commercially obtained from Sigma Laboratories (CAS No. 1115–70–4, Sigma-Aldrich).

### Experimental design

Forty adult specimens (males and females) of *Astyanax lacustris*, weighing approximately 9–11 g and measuring 10–15 cm in length, were obtained from a local fish breeding facility. The fish were acclimated in 100 L glass aquariums containing dechlorinated water, maintained at room temperature (25–28 °C) under a natural photoperiod (12:12 h light/dark cycle) with constant aeration. The animals were fed a standard diet consisting of commercial feed formulated for small fish (Alcon® Basic Fish Food, Camboriú, Santa Catarina, Brazil). The fish were acclimated for 10 days in the Fish Farming and Experimentation Vivarium at the State University of Maringá (UEM), Maringá, Paraná, Brazil.

Throughout the experiment, water quality parameters were carefully monitored to ensure compliance with standard recommendations. Water temperature was maintained between 25 and 28 °C, pH ranged from 7.2 to 7.5, ammonia concentration remained below 0.02 mg/L, nitrite levels were maintained below 0.1 mg/L, and dissolved oxygen was consistently sustained at saturation levels between 7 and 8 mg/L.

After the acclimation period, the fish were randomly divided into four groups: one control group (maintained in dechlorinated water only) and three treatment groups exposed to metformin at concentrations of 50 μg/L, 100 μg/L, and 10,000 μg/L for 90 days. A total of 20 fish (five individuals per group, including the control) were used for erythrocyte morphological biomarker evaluation and the comet assay. For flow cytometry analysis, an additional 20 fish were allocated similarly, with five fish assigned to each treatment condition (three metformin concentrations) and five fish to the control groups (negative and positive controls).

The concentrations of 50 μg/L and 100 μg/L metformin were selected based on levels reported in surface waters from different aquatic environments, whereas the concentration of 10,000 μg/L corresponds to the No Observed Effect Concentration (NOEC) established for zebrafish, *Danio rerio*, in chronic toxicity tests (≥ 7.8 mg/L) (Moermond and Smit 2016). To minimize metformin biotransformation during exposure, both the water and drug solutions were renewed every 48 h, considering the known stability of metformin (Sharma et al. 2010).

At the end of each treatment period, fish were anesthetized by immersion in water containing clove oil (diluted in alcohol) at a concentration of 5 mg/L, according to Inoue et al. (2005).

### Cellular morphological changes in erythrocytes

Cellular morphological changes were analyzed following the methodology described by Hooftman and De Raat (1982). After anesthesia, approximately 10 μL of peripheral blood was collected from the caudal vein of each specimen using a heparinized syringe to prepare blood smears. The smears were fixed in 100% ethanol for 20 min and stained with 5% Giemsa diluted in phosphate buffer (Sorensen, pH 6.8) for 10 min. A total of 2,000 erythrocytes per individual were analyzed, resulting in 10,000 cells per group (control and treatment groups).

The observed erythrocyte abnormalities were classified into erythrocyte cellular abnormalities (ECA) and erythrocyte nuclear abnormalities (ENA), according to the criteria proposed by Carrasco et al. (1990), Barni et al. (2007), Sayed et al. (2014), and Cherednichenko et al. (2024). Blood smear analysis and image acquisition were performed using a photomicroscope (BX51, Olympus) at 1000 × magnification.

### Comet assay

The comet assay was performed using peripheral blood erythrocytes following the protocol established by Speit and Hartmann (1999). Approximately 10 µL of blood was collected from the caudal vein of 10 specimens of *A. lacustris* using a heparinized syringe and diluted in 1 mL of fetal bovine serum. For microscopic analysis, slides were prepared by mixing 10 μL of the cell suspension with 120 μL of low-melting-point agarose at 37 °C. Subsequently, the slides were incubated in a lysis solution composed of 1 mL Triton X-100, 10 mL DMSO, and 89 mL of stock lysis solution containing 2.5 M NaCl, 100 mM EDTA (ethylenediaminetetraacetic acid), 10 mM Tris (pH 10), 2.5 M NaOH, and 1% N-laurylsarcosina. The slides were maintained in the dark at 8 °C for 1 h.

Following lysis, the slides were placed in an electrophoresis chamber and immersed in alkaline buffer solution (0.3 N NaOH, 1 mM EDTA, pH > 13) for 20 min to allow DNA unwinding under chilled conditions (8 °C). Electrophoresis was subsequently performed for 20 min at 25 V and 300 mA, with an electric field strength of 1 V/cm. The slides were then neutralized in 0.4 M Tris for 10 min, fixed in absolute ethanol for an additional 10 min, and stained with ethidium bromide (2 µg/mL).

Analyses were performed using an epifluorescence microscope (Zeiss Axioskop) at 400 × magnification, with 100 nucleoids evaluated per fish. DNA damage was visually classified into four categories according to DNA fragment migration patterns. The total number of DNA strand breaks was quantified in arbitrary units (AUT), ranging from 0 to 300, using the following equation: AUT = (N0 × 0) + (N1 × 1) + (N2 × 2) + (N3 × 3), where Ni represents the number of nuclei classified in each category (Collins and Dušinská 2002; Cestari et al. 2004).

### Evaluation of plasma membrane integrity by flow cytometry

Cell viability was assessed by flow cytometry according to the methodology described by Volpato et al. (2018). Approximately 20 µL of whole blood was collected from the caudal vein using a heparinized syringe from five fish per metformin treatment, as well as from positive and negative controls (the latter consisting of untreated blood samples). A positive control using digitonin was included to verify the reliability and feasibility of the method.

The heparinized blood samples were washed with 1 × PBS (phosphate-buffered saline) and stained with propidium iodide (PI) at a dilution of 10⁻^4^ for 5 min. PI is a nucleic acid marker that does not penetrate intact plasma membranes. A total of 10,000 cells per individual were analyzed. Data acquisition was performed using a BD FACSCalibur flow cytometer, and analyses were conducted using BD CellQuest Pro software, with a detection wavelength ranging from 560 to 580 nm (FL2 detector).

### Statistical analysis

After assessing data normality, the biological parameters, including nuclear and cellular morphological alterations, comet assay results, and flow cytometry data, were analyzed by the one-way ANOVA test. Tukey’s multiple comparison post hoc test was performed using GraphPad Prism version 5.0. A significance level of 5% (*p* < 0.05) was adopted for all analyses.

## Results

During the experiment, no significant mortality or abnormal behavior was observed in metformin-treated fish compared with the untreated control group, indicating that the experimental conditions were maintained at sublethal levels.

### Morphological changes in erythrocytes after metformin exposure

**Fig. 1:**
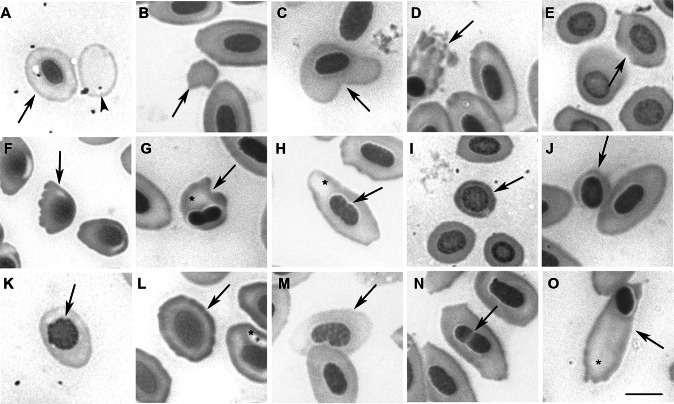
Photomicrograph of morphological changes in *Astyanax lacustris* erythrocytes after treatment with metformin; (**A**) normal erythrocytes (arrow), erythroplastid (arrowhead); (**B**) schizocyte; (**C**) cytoplasmic constriction; (**D**) and (**E**) cells with membrane rupture; (**F**) acanthocyte; (**G**) acanthocyte with lobed nucleus; (**H**) eightshaped nucleus; (**I**) immature cell; (**J**) macronucleus; (**K**) irregular nucleus; (**L**) echinocyte; (**M**) notched; (**N**) binucleated cell; (**O**) peripheral nucleus. (*) represent cytoplasmic vacuoles and arrows indicate the abnormalities. Bar = 10 µm

Analysis of erythrocytes from *A. lacustris* specimens revealed that the control group exhibited morphological characteristics typical of healthy cells, including an oval shape and the presence of a centrally located nucleus (Fig. 1A). In contrast, fish exposed to metformin displayed a variety of abnormalities, including cytoplasmic and membrane alterations, classified as erythrocyte cellular abnormalities (ECA), as well as nuclear alterations, classified as erythrocyte nuclear abnormalities (ENA), as illustrated in Fig. 1.

Table 1 summarizes the different types and frequencies of erythrocyte alterations observed, expressed as absolute values, totaling 14 distinct abnormalities. The erythrocyte abnormalities illustrated in Fig. 1 included cytoplasmic vacuolization (Fig. 1G and H, L, O), membrane invagination (Fig. 1C, accompanied by cytoplasmic constriction), and membrane rupture (Fig. 1D and E), particularly in fish exposed to the highest metformin concentrations (100 and 10,000 µg/L). These alterations showed significant differences compared with the control group (Table 1).

**Table 1:** Absolute values of morphological changes in *Astyanax lacustris* erythrocytes after metformin treatment, (2,000 cells were analyzed by fish)

	Abnormalities	Control	Treatments 50	(µg/L) 100	10,000
Abnormalities in the plasma membrane and/or cytoplasm (ECA)	erythroplastids	20	69*	1	14
	schizocyte	2	0	0	0
	membrane invagination	13	25	273	928*
	membrane rupture	0	12	6	76*
	acanthocyte	1	21	4	117
	echinocyte	1	0	39	0
	cytoplasmic vacuole	20	0	1342*	1268*
Abnormalities in the nucleus of erythrocytes (ENA)	eightshaped	0	0	6	0
	immature erythrocyte	9	2	74	3
	macronucleus	5	4	54	113
	irregular nucleus	0	0	107	1
	notched	0	7	3	77
	binucleate	2	2	2	6
	peripheral nuclei	8	44	5	1
Total of the changes		79	184	1916*	2604*

^*^Significantly different to control group (p < 0.05)

Additional abnormalities affecting erythrocyte morphology, including erythroplastids (Fig. 1A), schizocyte (Fig. 1B), acanthocytes (Fig. 1F), and echinocytes (Fig. 1L), exhibited variable frequencies among treatment groups relative to the control group. Notably, erythrocytes exposed to 50 µg/L metformin exhibited significantly higher frequencies of erythroplastids (enucleated cellular forms; Fig. 1A) compared with the control group.

Several erythrocyte nuclear abnormalities (ENA) were also identified, including eight-shaped nuclei (Fig. 1H), macronuclei (Fig. 1J), immature erythrocytes (Fig. 1I), irregular nuclei (Fig. 1K), notched nuclei (Fig. 1M), and binucleated cells (Fig. 1N). Although the frequency of these abnormalities varied among experimental groups, no statistically significant differences were detected (Table 1). Peripheral nuclear displacement, irregular nuclei, and macronuclei were the most frequent abnormalities at concentrations of 50 µg/L, 100 µg/L, and 10,000 µg/L, respectively.

**Fig. 2:**
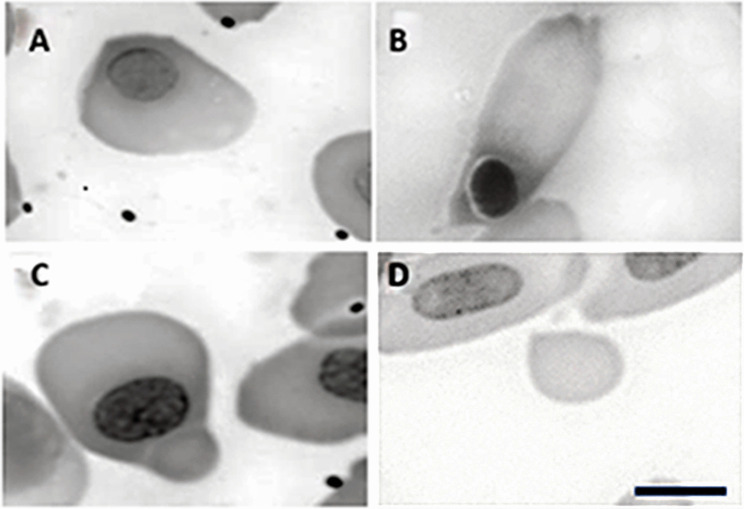
Photomicrograph of *Astyanax lacustris* erythrocytes exposed to metformin treatments showing changes related to the denucleation process; (**A**) and (**B**) peripheralization of the nucleus; (**C**) formation of the erythroplastid with cytoplasmic constriction; (**D**) erythroplastid (cell without a nucleus). Bar = 5 µm

Among the cytoplasmic and nuclear abnormalities observed in erythrocytes, a possible denucleation process was noteworthy, characterized by nuclear displacement toward the cell periphery and observed in numerous cells (Fig. 1O; Fig. 2). Cytological preparations from fish exposed to the three metformin concentrations revealed erythrocytes exhibiting peripheral nuclear displacement (Fig. 2A and B). Additional alterations were also identified, including cells with cytoplasmic constriction (Fig. 2C) and erythroplastid formation, characterized by enucleated cells (Fig. 2D). Therefore, these changes were interpreted as possible stages in the cell denucleation process.

### Evaluation of plasma membrane integrity by flow cytometry

Propidium iodide (PI) is a membrane-impermeable nucleic acid stain. Cells with compromised plasma membrane integrity allow PI entry and binding to nucleic acids, resulting in high fluorescence intensity, represented in the upper left (UL) and upper right (UR) quadrants of the histograms (Fig. 4), as confirmed by BD CellQuest Pro software. In contrast, cells located in the lower left (LL) and lower right (LR) quadrants exhibited low fluorescence intensity and were considered viable, indicating preserved plasma membrane integrity.

**Fig. 3:**
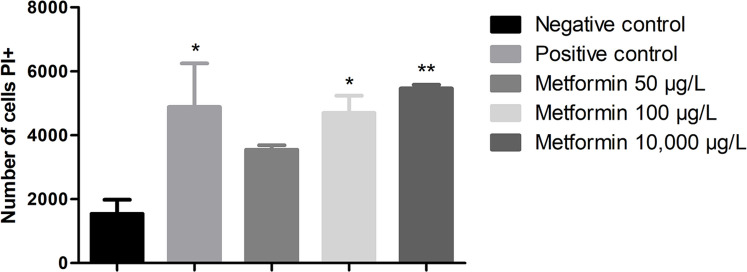
Number of cells PI.^+^ measured by flow cytometry in *Atyanax lacustris* blood cells, after metformin treatment and control groups; digitonin was used in the positive control group; 10,000 cells per fish were analyzed (*n* = 20). *Significantly different to control group (*p < 0,05 and ** *p* < 0,005)

Analysis of 10,000 blood cells per fish from *A. lacustris* revealed an increase in the number of PI-labeled cells in all metformin treatment groups compared with negative control. Digitonin, used as a positive control due to its membrane-permeabilizing properties, induced a marked increase in nonviable cells, as evidenced by elevated fluorescence levels (Fig. 3, Table S1). Similar effects were observed in fish exposed to metformin concentrations of 100 and 10,000 µg/L (Fig. 3, Table S1).

**Fig. 4:**
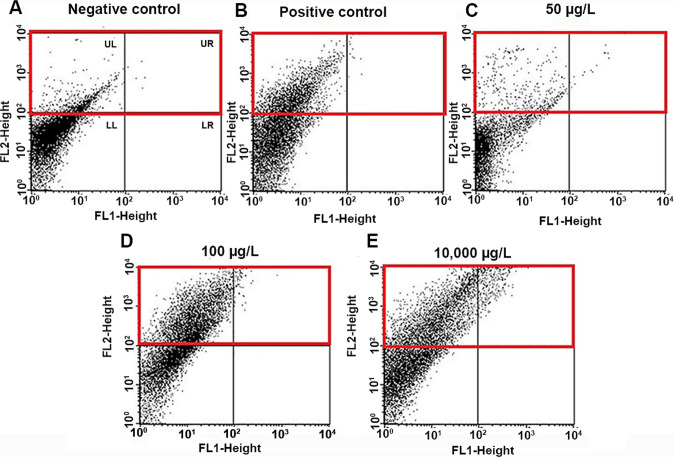
Histogram representing the data obtained from the flow cytometer of whole blood samples of *Astyanax lacustris*, after exposure to different concentrations of metformin, including control groups, negative (**A**) and positive using digitonin (**B**); (**C**, **D** and **E**) represent the number of cells that emitted high fluorescence (quadrants highlighted in red). The upper left (UL) and right (UR) quadrants showed high fluorescence; cells in the lower left (LL) and right (LR) quadrants were considered viable cells, as they showed low fluorescence

Fluorescence intensity detected by flow cytometry was quantified as a percentage, considering cells distributed in the upper right (UR) and upper left (UL) quadrants for each treatment group. Fish exposed to digitonin and metformin were compared with the untreated control group (Fig. 4A). Cells exposed to digitonin exhibited a 217% increase in fluorescence (Fig. 4B). Fish exposed to 50 µg/L metformin (Fig. 4C) showed a 130% increase, whereas the 100 µg/L treatment group (Fig. 4D) exhibited a 205% increase in fluorescence intensity. Notably, fish exposed to 10,000 µg/L metformin (Fig. 4E) displayed the highest fluorescence percentage, exceeding 255%. Histogram profiles demonstrated a clear dose-dependent effect in fish exposed to metformin compared with the negative control group (Fig. 4).

### Comet assay

The comet assay revealed DNA damage in erythrocytes of *A. lacustris*, classified as damage types 1 and 2. DNA damage scores are presented in Table 2. Significant genotoxic effects were observed at the highest metformin concentrations, specifically 100 and 10,000 µg/L. As metformin concentration increased, a corresponding increase in DNA damage was observed. However, from 100 µg/L onward, a plateau effect was detected, with damage levels remaining relatively stable up to the concentration of 10,000 µg/L.

**Table 2:** Frequency of DNA damage in *Astyanax lacustris* erythrocytes after exposure to metformin. One hundred nucleoids from each fish were analyzed

Concentrations	Specimens	Damage classes						Mean ± standard deviation of scores
		**0**	**1**	**2**	**3**	**4**	scores	
Control	1	99	1	0	0	0	1	2,2 ± 1,78
	2	95	5	0	0	0	5	
	3	99	1	0	0	0	1	
	4	99	1	0	0	0	1	
	5	97	3	0	0	0	3	
50 µg/L	6	91	8	1	0	0	10	9,4 ± 2,19
	7	89	9	2	0	0	13	
	8	92	8	0	0	0	8	
	9	93	6	1	0	0	8	
	10	93	6	1	0	0	8	
100 µg/L	11	93	6	1	0	0	8	11,2 ± 4,54*
	12	95	5	0	0	0	5	
	13	89	8	3	0	0	14	
	14	88	8	4	0	0	16	
	15	89	9	2	0	0	13	
10,000µg/L	16	89	10	2	0	**0**	14	11,8 ± 4,76*
	17	86	13	3	0	0	19	
	18	93	5	2	0	0	9	
	19	93	7	0	0	0	7	
	20	90	10	0	0	0	10	

^*^Significantly different to control group (p < 0.05)

## Discussion

Hematological parameters serve as important indicators of fish health and are widely used to assess the toxicity of various aquatic contaminants (Arnaudov and Arnaudova 2023), including pharmaceuticals (Guiloski et al. 2017; Sayed et al. 2022; Taher et al. 2022). Studies involving different fish species and evaluating parameters such as erythrocyte and leukocyte counts, as well as hemoglobin concentrations, have demonstrated substantial alterations following pharmaceutical exposure. For example, significant hematological changes have been reported in *Rhamdia quelen* exposed to paracetamol (Guiloski et al. 2017), African catfish *Clarias gariepinus* exposed to dexamethasone (Sayed et al. 2022), and *C. gariepinus* exposed to metformin (Taher et al. 2022). Additional hematological disturbances in fish include erythrocyte abnormalities such as cytoplasmic and nuclear inclusions, cellular deformation, amitosis, hemolysis, and alterations in cell size and staining properties (Witeska 2013; Arnaudov and Arnaudova 2023; Cherednichenko et al. 2024). Erythrocyte morphology has proven to be more sensitive to certain toxicants than conventional hematological parameters, such as erythrocyte count, hemoglobin concentration, and hematocrit, allowing the detection of cellular abnormalities even when core hematological indices remain unchanged (Witeska et al. 2022). Despite the growing number of studies investigating the effects of metformin in fish species, a considerable gap remains regarding its effects on erythrocyte morphology.

In the present study, evaluation of erythrocyte cellular abnormalities (ECA) in *A. lacustris* revealed a marked occurrence of cytoplasmic vacuoles following exposure to metformin concentrations of 100 and 10,000 µg/L. Barbieri et al. (2024) similarly reported a significant frequency of cytoplasmic vacuoles in erythrocytes of *Danio rerio* chronically exposed to metformin concentrations of 150 and 10,000 µg/L. These authors suggested that cytoplasmic vacuoles may have an autophagic origin associated with oxidative stress induced by the drug, functioning as a protective mechanism to ensure cell survival or, depending on the severity of the damage, promoting cell death. Cellular vacuolization is a common response to exposure to pharmaceutical agents and other chemical contaminants, acting as a cell-autonomous defense mechanism against toxic compounds. Although this process may be reversible following the removal of the toxicant, prolonged exposure can make it irreversible, ultimately resulting in cell death (Aki et al. 2012). In vertebrates possessing nucleated erythrocytes, including fish, amphibians, and reptiles, the presence of cytoplasmic vacuoles has been associated with the removal of extracellular toxins and may indicate the onset of pathological processes within the organism (Cherednichenko et al. 2024).

Cytoplasmic vacuolization has also been described in mammalian cells exposed to pathogens, including viruses and bacteria, as well as to several natural and synthetic low-molecular-weight compounds. This phenomenon is frequently associated with cell death, although the underlying mechanisms remain incompletely understood (Shubin et al. 2016). Several toxicological studies in fish have reported the occurrence of cytoplasmic vacuoles in erythrocytes following chemical exposure. Examples include *Labeo rohita* exposed to the herbicide butachlor (Ghaffar et al. 2015), *Channa punctata* exposed to the organochlorine pesticide lindane (Bhattacharjee and Das 2017), and common carp *Cyprinus carpio* exposed to fipronil (phenylpyrazole) (Ghaffar et al. 2018). Therefore, the high frequency of cytoplasmic vacuolization observed in the present study likely represents a response to metformin exposure and may indicate the development of more severe pathological effects.

Other erythrocyte cellular abnormalities (ECA), including membrane invaginations and membrane rupture, which occurred at significantly higher frequencies at elevated metformin concentrations, suggest possible alterations in the lipid composition of the plasma membrane, potentially impairing membrane fluidity (Muller et al. 1997; Bardhan et al. 2024). Such changes may compromise membrane stability, leading to cellular damage and consequent morphological alterations in blood cells (Sayed et al. 2023). Analysis of plasma membrane-related erythrocyte abnormalities, together with flow cytometry data, provided additional evidence of cellular impairment in *A. lacustris* exposed to metformin. Flow cytometry histograms revealed elevated fluorescence intensity, indicating compromised membrane integrity and a dose-dependent response to metformin exposure. Propidium iodide is an effective marker of plasma membrane integrity because it binds to nucleic acids only after membrane disruption (Volpato et al. 2018), corroborating the microscopic evidence of erythrocytes exhibiting membrane rupture and invaginations observed in this study. Furthermore, flow cytometric analyses indicated a significant increase in the number of non-viable cells following metformin exposure. As an adaptive response, as the proportion of non-viable cells increases, the remaining circulating erythrocytes may undergo morphological and volumetric adjustments to maintain adequate oxygen transport to tissues (Witeska 2013). Under adverse environmental conditions, fish may activate compensatory mechanisms to maintain oxygen supply, including increased erythrocyte and hemoglobin production, amitotic erythrocyte division, stimulation of hematopoiesis, and mobilization of erythrocytes from hematopoietic and storage organs such as the kidney and spleen (Witeska 2013; Arnaudov and Arnaudova 2023).

Abnormally shaped erythrocytes in fish, collectively referred to as poikilocytosis, include forms such as acanthocytes, echinocytes, dacrocytes, and schistocytes, among others, and are well documented in aquaculture and toxicological studies. These abnormalities are associated with multiple factors, including pathological, nutritional, toxicological, and environmental stressors (Bardhan et al. 2024). Acanthocytes, characterized by irregularly distributed membrane spicules, were more frequently observed, although not significantly, in *A. lacustris* exposed to 10,000 µg/L metformin. Acanthocytes and echinocytes have been reported in numerous fish species, frequently occurring at high percentages (≥ 80%) under toxicological conditions (Bardhan et al. 2024). Soliman and Sayed (2020) documented a significant increase in poikilocytosis, including several membrane and nuclear abnormalities, in juvenile tilapia *Oreochromis niloticus* exposed to tramadol. Some abnormalities reported by these authors are comparable to those identified in the present study, including acanthocytes, schistocytes, and eccentric nuclei. Poikilocytosis may result from internal physiological disturbances, environmental stressors, or genetic abnormalities, including mutations affecting erythroid cytoskeletal proteins. However, interpretation of poikilocytic responses remains complex due to the multiple interacting factors capable of influencing erythrocyte morphology in fish exposed to xenobiotic compounds (Bardhan et al. 2024).

It is important to note that some erythrocyte abnormalities, including acanthocytes, erythroplastids, peripheral nuclei, immature erythrocytes, and irregular nuclei, observed in the present study did not exhibit a clear linear dose-dependent relationship following metformin exposure. Instead, certain alterations occurred more frequently at low, intermediate, or high concentrations. Such heterogeneous responses have been commonly reported in hematological biomarkers of fish exposed to xenobiotic compounds and may be associated with complex biological mechanisms, including compensatory physiological adjustments, cellular adaptation, fluctuations in oxidative stress, and the removal of damaged cells from circulation (Witeska 2013; Khatun et al. 2021; Arnaudov and Arnaudova 2023).

Erythrocyte nuclear abnormalities (ENA) in *A. lacustris* occurred less frequently than cytoplasmic and plasma membrane alterations (ECA) following metformin exposure. Nevertheless, the occurrence of different types of nuclear abnormalities may have important biological implications for erythrocyte viability. The frequencies of macronuclei and notched nuclei were higher in fish exposed to the highest metformin concentration (10,000 µg/L), whereas irregular nuclei were more frequently observed in fish exposed to 100 µg/L metformin. Although these alterations did not differ significantly from the control group, they suggest structural impairment of the nucleus induced by metformin exposure. Nuclear abnormalities, including nuclear budding, nuclear envelope invaginations, nucleoplasmic bridges, protrusions, binucleated erythrocytes, amitosis, and tail-shaped nuclei, among others, may indicate not only degenerative cellular processes but also possible chromosomal aberrations (Cherednichenko et al. 2024).

Immature erythrocytes were identified in the peripheral blood of *A. lacustris*, occurring more frequently in fish exposed to 100 µg/L metformin. Although this difference was neither statistically significant nor dose-dependent, its occurrence may still be biologically relevant. In teleost fish, the head kidney constitutes the primary erythropoietic organ, and the weak barrier between hematopoietic tissue and the circulatory system facilitates the release of immature erythrocytes into peripheral blood. These immature erythrocytes are typically rounder in shape and possess proportionally larger nuclei, often representing more than 10% of circulating erythrocytes (Witeska 2013; Arnaudov and Arnaudova 2023). Consequently, erythrocyte damage induced by environmental stressors may be compensated by the release of newly formed cells from hematopoietic organs such as the cephalic kidney or erythrocyte storage organs such as the spleen, thereby increasing the frequency of immature erythrocytes in circulation (Witeska 2013). Therefore, the detection of immature erythrocytes in *A. lacustris* exposed to metformin may reflect an adaptive physiological response to drug-induced stress.

Nuclear alterations may serve as biomarkers of DNA damage and, consequently, indicate genotoxic effects (Bhattacharjee and Das 2017). In the present study, the mean comet assay scores recorded in metformin-exposed groups exceeded those of the control group, indicating a concentration-dependent increase in DNA damage. Notably, significant genotoxic effects were observed at the highest metformin concentrations (100 and 10,000 μg/L). The genotoxic potential of metformin has previously been investigated using chromosomal aberration (CA), micronucleus (MN), and comet assays. Amador et al. (2012) reported that exposure of CHO-K1 hamster ovary cells to metformin concentrations of 114.4 and 572 mg/mL did not induce genotoxic effects in either CA or MN assays. However, significant DNA damage was detected in the comet assay after 24 h of exposure at both concentrations. Similarly, Sant'Anna et al. (2013) demonstrated that exposure of human lymphocyte cultures to metformin concentrations of 12.5, 25, and 50 mM did not significantly alter the frequency of numerical or structural chromosomal aberrations or micronuclei, suggesting the absence of detectable mutagenic effects under the tested conditions.

In contrast, Yuzbasioglu et al. (2020) investigated the genotoxic potential of metformin using chromosomal aberration (CA), micronucleus (MN), and sister chromatid exchange (SCE) assays in human peripheral lymphocytes, as well as the comet assay in isolated lymphocytes, after exposure to different concentrations (12.5, 50, 75, 100, and 125 mg/mL) for 24 or 48 h. The authors reported dose- and exposure time-dependent effects. Specifically, metformin increased the frequency of CA after 48 h of exposure, induced SCE formation in all treated groups, and increased MN frequency at the highest concentration after 24 h. In addition, DNA damage was evidenced by increased comet tail length across all treatment groups. These apparently contradictory findings suggest that metformin-induced genotoxicity may be modulated by dose, exposure duration, and tissue type, and that some compounds may exhibit either pro-genotoxic or anti-genotoxic effects depending on the biological context (Amador et al. 2012).

Environmental contaminants, including pharmaceuticals, may directly damage DNA or indirectly induce oxidative stress through the generation of reactive oxygen species (ROS), thereby affecting lipids, proteins, and nucleic acids (Guiloski et al. 2017; Biller and Takahashi 2018; Sayed et al. 2023; Sibiya et al. 2023). Similar effects have been reported in *D. rerio* embryos exposed to metformin concentrations ranging from 1 to 100 µg/L, which exhibited increased oxidative stress biomarkers (Elizalde-Velázquez et al. 2021). Likewise, exposure of *Labeo rohita* to metformin concentrations of 40 and 80 µg/L resulted in increased production of reactive oxygen species and free radicals, particularly at the highest concentration, ultimately leading to substantial DNA damage in genotoxicity assays (Sibiya et al. 2023). Therefore, the DNA damage observed in *A. lacustris* erythrocytes in the present study may be associated with oxidative stress mechanisms induced by metformin exposure.

An intriguing finding observed in erythrocytes of *A. lacustris* exposed to metformin was nuclear displacement toward the cell periphery. These erythrocytes exhibited peripheral nuclear localization associated with membrane invagination and erythroplastid formation, suggesting a possible denucleation process. Similar alterations were described by Barni et al. (2007) based on observations of erythrocytes from peripheral blood of *Rana esculenta*, including both adults and tadpoles inhabiting polluted rice fields. In tadpoles, the authors reported erythrocytes exhibiting marked cytoplasmic constriction and nuclear displacement toward the cell periphery, suggesting a denucleation process, a term proposed by Barni et al. (2007), accompanied by erythroplastid formation (anucleated circulating erythrocytes). In addition, those authors reported erythrocytes undergoing division, characterized by mitotic and partially amitotic-like figures displaying symmetrical or asymmetrical nuclear and cytoplasmic segmentation.

No evidence of mitotic or amitotic-like figures was observed in the present study. Although amitosis is considered uncommon, it has been documented in fish erythrocytes, including triploid rainbow trout *Oncorhynchus mykiss* (Wang et al. 2010) and round goby *Neogobius melanostomus* subjected to experimental hypothermia (Soldatov et al. 2020). Nevertheless, the occurrence of peripheral nuclear displacement, cytoplasmic constriction, and erythroplastid formation observed in *A. lacustris* aligns with the findings of Barni et al. (2007), supporting the hypothesis of nuclear extrusion. Moreover, a notable frequency of erythroplastids was detected at the lowest metformin concentration (50 µg/L), suggesting that denucleation may represent a cellular response to drug-induced stress.

Interestingly, stronger biological responses at low contaminant concentrations have frequently been reported for endocrine-disrupting chemicals (EDCs) (Vandenberg et al. 2012). Although the limited number of tested concentrations in the present study does not allow for the characterization of a true non-monotonic dose–response relationship, the pronounced occurrence of erythroplastids at the lowest metformin concentration (50 µg/L) may reflect complex physiological responses associated with the endocrine-disrupting effects of metformin previously reported in fish. Among the toxic effects associated with metformin exposure in fish are intersex conditions and endocrine disruption. Such effects have been demonstrated in adult fathead minnows *Pimephales promelas* (Niemuth et al. 2015) and medaka *Oryzias latipes* (Lee et al. 2019) exposed to metformin at concentrations as low as 40 µg/L.

During erythropoiesis in vertebrates, erythroid precursor cells undergo progressive nuclear condensation; however, complete nuclear extrusion occurs only in mammals, a process known as enucleation (Ji et al. 2011; Mei et al. 2020). This process begins with migration of the nucleus toward one side of the cytoplasm, establishing cellular polarity, followed by rapid nuclear extrusion and subsequent detachment from the developing reticulocyte, which then undergoes maturation (Mei et al. 2020). In contrast, fish erythrocytes retain their nuclei throughout maturation and maintain structural integrity through three major filament systems: a membrane-associated actin–spectrin cytoskeleton, intermediate filaments, and microtubules (Verde et al. 2011). Therefore, the occurrence of erythroplastids and peripheral nuclei in *A. lacustris* erythrocytes may indicate a pathological disruption of normal erythrocyte homeostasis induced by chronic metformin exposure.

In addition to oxygen transport, fish erythrocytes can participate in innate immune defense mechanisms through an evolutionarily conserved process known as ETosis (Rinaldi et al. 2023). Initially described in human neutrophils, ETosis involves the release of extracellular traps (ETs), composed of chromatin-associated DNA, antimicrobial peptides, and enzymes capable of immobilizing and eliminating pathogens (Wartha and Henriques-Normark 2008). This process involves extracellular DNA extrusion and has subsequently been documented in other innate immune cells, including eosinophils, mast cells, monocytes, macrophages, basophils, microglia, and dendritic cells, across diverse organisms such as mammals, birds, insects, and plants (Koh et al. 2023).

In fish, ETosis-like responses have been described in erythrocytes of *Danio rerio* and *Salmo salar*, termed Fish Erythrocyte Extracellular Traps (FEETs), following exposure to chemical and physiological stimuli (Rinaldi et al. 2023). Based on these findings, the nuclear alterations observed in *A. lacustris* erythrocytes, culminating in erythroplastid formation and possible nuclear extrusion, may represent a defense response against prolonged exposure to chemical contaminants, particularly considering the chronic exposure period to metformin (90 days). However, because no specific molecular or ultrastructural analyses were conducted in the present study, this interpretation remains speculative and should be investigated further using targeted methodologies.

## Conclusion

This study clearly demonstrates that chronic exposure to metformin at concentrations of 50, 100, and 10,000 µg/L induces significant alterations in erythrocytes of *A. lacustris*. Notably, the highest metformin concentration tested (10,000 µg/L), corresponding to the “No Observed Effect Concentration” (NOEC) previously established for *D. rerio*, highlights the need to reassess this threshold considering the sensitivity of other fish species.

The morphological abnormalities observed in erythrocytes, particularly those affecting the plasma membrane and cytoplasm, as well as nuclear shape, indicate impaired membrane integrity and altered membrane permeability. These findings were further supported by flow cytometry analyses, reinforcing the hypothesis that pathological cellular damage induced by metformin may compromise erythrocyte viability and potentially culminate in cell death. Furthermore, cytopathological alterations, including peripheral nuclear displacement and the consequent formation of erythroplastids, suggest a possible denucleation process, raising important concerns, particularly when considered alongside the occurrence of DNA damage.

Taken together, the integrated use of erythrocyte morphology, flow cytometry, and comet assay demonstrated that metformin exerts marked cytotoxic and genotoxic effects in *A. lacustris*. The findings presented here contribute to advancing hematological and ecotoxicological knowledge in fish by improving our understanding of erythrocyte structure, physiology, and responses to environmental contaminants. Ultimately, chronic metformin exposure may represent a relevant long-term health risk to aquatic organisms, emphasizing the importance of monitoring pharmaceutical contamination in aquatic ecosystems.

## Supplementary Information

Below is the link to the electronic supplementary material.

Supplementary file1 (DOCX 14 KB)

## Data Availability

All datasets generated or analyzed during this study are included in this article.
