# Supplementary material for: Structural abnormalities in erythrocytes and a potential denucleation process induced by metformin, compromising cell viability in specimens of Astyanax lacustris (Pisces, Characidae)
Source: Environ Sci Pollut Res Int. 2026 Jul 23;33(24):12362–75. doi: 10.1007/s11356-026-38072-5 (PMC13468855; doi:10.1007/s11356-026-38072-5)
Supplement: Supplementary file 1 [file 11356_2026_38072_MOESM1_ESM.docx]

**Table S1.** Means and standard deviations of the cell frequencies obtained by flow cytometer of whole blood samples of *Astyanax lacustris*, after treatment with metformin and control groups.

| **Treatment** | **Mean ± SD** |
| --- | --- |
| Negative control | 1,538.00 ± 772.94 |
| Positive control* | 4,885.50 ± 2,358.07 |
| Metformin 50 µg/L | 3,542.75 ± 248.96 |
| Metformin 100 µg/L* | 4,692.75 ± 940.89 |
| Metformin 10,000 µg/L** | 5,462.00 ± 194.44 |

*Significantly different to control group (*p < 0,05 and ** p < 0,005)
